# Supplementary material for: Human Brain Shows Recurrent Non-Canonical MicroRNA Editing Events Enriched for Seed Sequence with Possible Functional Consequence
Source: Noncoding RNA. 2020 Jun 2;6(2):21. doi: 10.3390/ncrna6020021 (PMC7345632; doi:10.3390/ncrna6020021)
Supplement: Supplementary file 1 [file ncrna-06-00021-s001.zip › ncrna-801776-suppl-final/Supplementary Figure S2.pptx]

## Slide 1
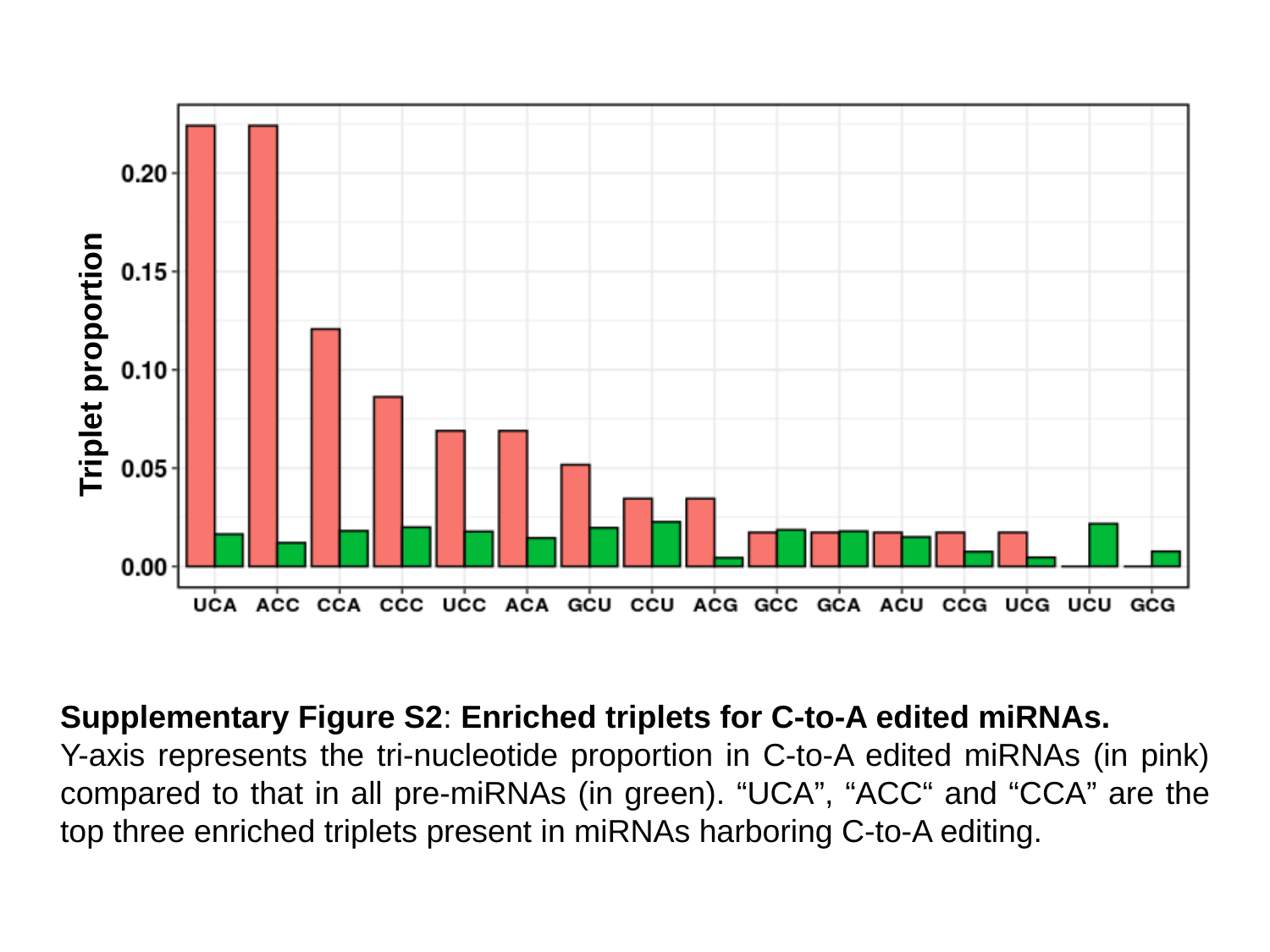

Triplet proportion
Supplementary Figure S2: Enriched triplets for C-to-A edited miRNAs.
Y-axis represents the tri-nucleotide proportion in C-to-A edited miRNAs (in pink) compared to that in all pre-miRNAs (in green). “UCA”, “ACC“ and “CCA” are the top three enriched triplets present in miRNAs harboring C-to-A editing.
