# Supplementary material for: Human Brain Shows Recurrent Non-Canonical MicroRNA Editing Events Enriched for Seed Sequence with Possible Functional Consequence
Source: Noncoding RNA. 2020 Jun 2;6(2):21. doi: 10.3390/ncrna6020021 (PMC7345632; doi:10.3390/ncrna6020021)
Supplement: Supplementary file 1 [file ncrna-06-00021-s001.zip › ncrna-801776-suppl-final/Supplementary Figure S3.pptx]

## Slide 1
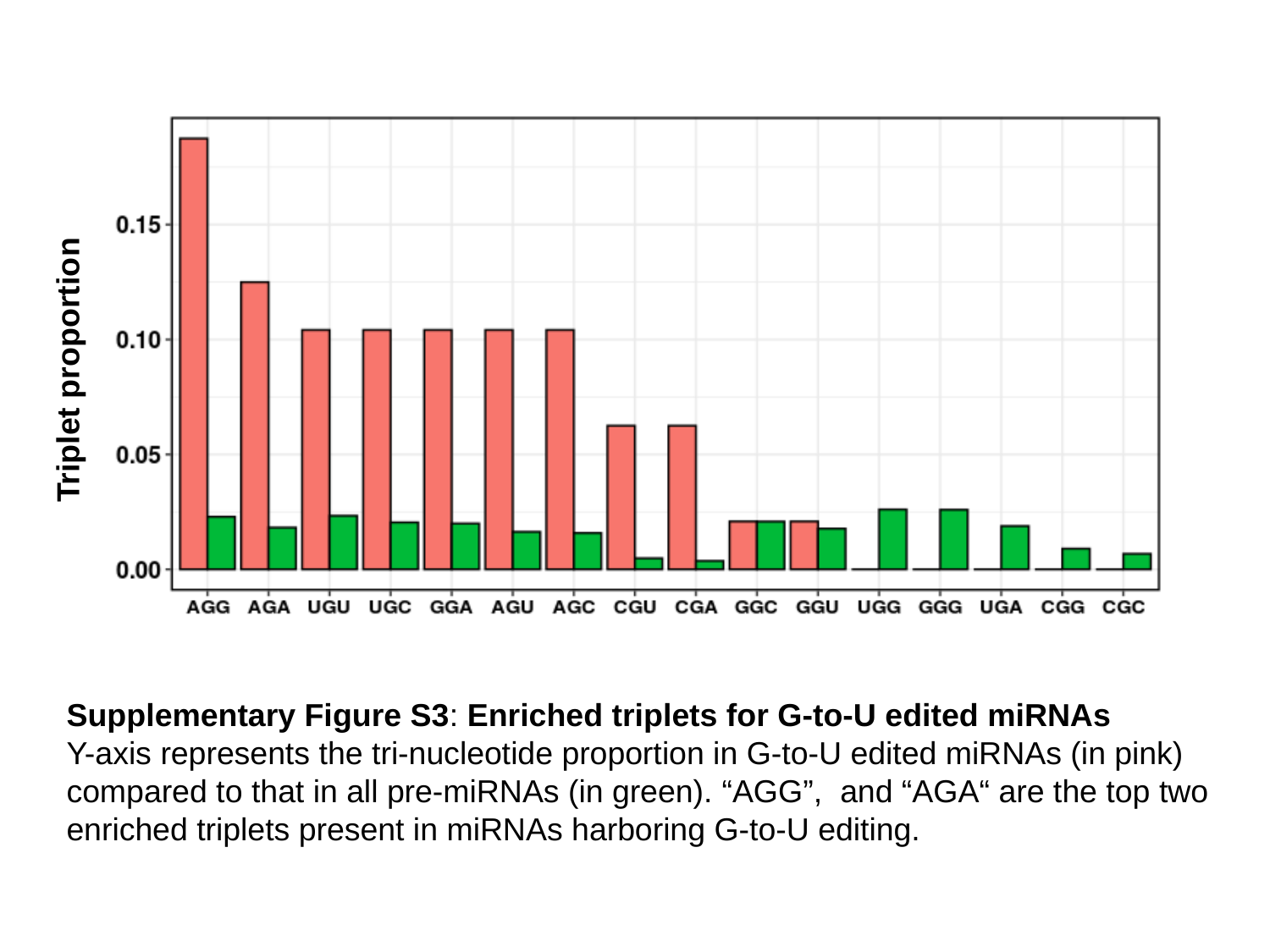

Triplet proportion
Supplementary Figure S3: Enriched triplets for G-to-U edited miRNAs
Y-axis represents the tri-nucleotide proportion in G-to-U edited miRNAs (in pink) compared to that in all pre-miRNAs (in green). “AGG”, and “AGA“ are the top two enriched triplets present in miRNAs harboring G-to-U editing.
